# Ethical dilemmas in narrative research: a review informed by Eastern wisdom traditions

**DOI:** 10.3389/frma.2025.1656083

**Published:** 2026-01-12

**Authors:** Rejina K. C., Niroj Dahal

**Affiliations:** Kathmandu University School of Education, Lalitpur, Nepal

**Keywords:** ethical dilemmas, narrative research, relational ethics, reflexivity, Eastern philosophy

## Abstract

Narrative research is intended to explore human experiences. However, there are ethical dilemmas that challenge researchers beyond formal protocols. This review examines 16 empirical studies (2014-2023) alongside insights from Eastern wisdom traditions, drawing on the experiences of three university faculty members who have employed narrative inquiry methodology in their graduate-level research to explore ethical dilemmas and shortcomings. This review identifies key recurring dilemmas in narrative research, including navigating informed consent, ensuring anonymity/confidentiality, managing power dynamics, mitigating emotional vulnerability, and respecting cultural sensitivity. The findings feature ethical integrity that relies on continuous reflexivity, relational ethics, and trust-building-principles reflected in Eastern concepts such as Dharma (i.e., righteous duties) and Karma (i.e., selfless actions). The study emphasizes the importance of context-sensitive ethical practices that prioritize participant dignity and the researcher's integrity. The article addresses the implications of creating ethical dilemmas in narrative research guidelines, provides ongoing ethical training, and promotes collaborative learning among researchers to enhance the trustworthiness of qualitative research in general and narrative research in particular.

## 1 Introduction

Ethical dilemmas in narrative research extend far beyond institutional review board approvals and formal consent procedures, manifesting as complex moral challenges that test researchers' judgment throughout the entire research process. Narrative research or inquiry in qualitative methodology traditions centers on human experiences, stories, meanings, and perspectives (Dahal, 2025; Dahal et al., 2024). It is for creating a unique ethical terrain in which researchers navigate tensions among methodological rigor and participants' rights, data-collection goals and relational responsibilities, and academic requirements and human dignity. At the same time, institutional ethical protocols provide essential frameworks that often present researchers with ethical dilemmas in the lived reality of conducting narrative research. Therefore, there is a need for contextual sensitivity, reflexive decision-making, and a philosophical grounding that goes beyond procedural compliance.

As researchers from the South Asian contexts\and having experience in narrative inquiry (see Dahal, 2022), we bring both insider perspectives on Eastern wisdom traditions and lived experiences of ethical challenges in qualitative research in general and narrative research. Our positions as faculty members at Tribhuvan University and the Kathmandu University School of Education shape our understanding of both Western ethical frameworks and Eastern wisdom traditions, influencing our interpretation of ethical dilemmas through cultural and professional lenses. This positionality enables us to examine how, while foundational, traditional Western bioethical principles may require integration with broader wisdom traditions that emphasize relational ethics, continuous reflexivity, and holistic approaches to human dignity. Thus, the complexity of ethical challenges in narrative research becomes apparent through the lived experiences of practicing researchers. Aligning with the above ethical dilemmas in narrative research or inquiry, we have considered the following reflections from three experienced narrative researchers who grappled with them firsthand:

*Maintaining all ethical considerations can sometimes limit us in ways that may not facilitate the exploration of the original lived stories of research participants. Yes, I do believe that ethical issues arise from data collection to interpretation and from writing the research report. If I have to share my experience, it is even vulnerable. I faced problems from framing the research questions to the final report. It was difficult during the interview; I was unable to collect pertinent stories due to some issues with the research question. I was unable to elicit a satisfactory response from the participants. Somewhere, I lost the trust of my participants because I was unable to inform them more effectively. Therefore, I was unable to explore the actual data at that time. It took me a long time to convince my participant to participate in another round of interviews. (R1, Field Data, December 2024)*

*I experienced difficulties in maintaining the complete anonymity of participants' profiles. Despite using a pseudonym, the originality and unique qualities of the participant may distort their anonymity. I had difficulty deciding whether to include the critical insight that participants underwent. The next challenge was to obtain a fake or exaggerated answer from a participant due to the presence of a recorder during the interview. Later, we created a good bond, and participants shared actual data. (R2, Field Data, December 2024)*

*For me, maintaining anonymity was a challenge, and I was informed that participants would benefit as well after being involved in the research. I was informed that it is a voluntary job. Here, I am not talking about monetary benefit, but rather it is about knowledge and experience. The next is all the theories that were in my mind during the interview, so I could not focus on my responsibility as a researcher. Format, timing, theories, management issues, and the participant context are some issues that created a sort of dilemma in my research journey. (R3, Field Data, January 2025)*

The above narratives demonstrate the multifaceted nature of ethical challenges in narrative research by revealing dilemmas that emerge at the intersection of methodological requirements and human relationships. In this regard, R1's experience features the delicate balance between ethical compliance and authentic data collection, demonstrating how procedural ethics can sometimes create barriers to genuine engagement. Moreover, R2's struggle with anonymity and authenticity reveals the tension between protecting participant identity and preserving the richness of narrative data. On the other hand, R3's reflection on the researcher's focus and the benefits to participants underscores the complexity of managing multiple ethical considerations simultaneously while maintaining research integrity. Drawing from these lived experiences, qualitative researchers engaged in narrative inquiry often encounter moral dilemmas that extend beyond traditional ethical protocols. These challenges include maintaining participant trust while adhering to formal procedures (see R1), ensuring genuine anonymity without compromising data authenticity (see R2), and balancing theoretical frameworks with relational responsibilities (see R3).

Furthermore, researchers face ongoing challenges in obtaining meaningful informed consent, managing power dynamics, addressing emotional vulnerability, and navigating cultural sensitivities throughout the research process. Despite adherence to institutional ethical protocols, researchers frequently find their judgment tested in ways that go beyond theoretical guidelines when balancing data-collection demands with the need to uphold participant autonomy and dignity. For instance, R1, R2, and R3 (three experienced narrative researchers) are university faculty members who employed narrative inquiry methodology in their graduate-level research and ongoing academic practice. Their reflections, grounded in substantial experience addressing ethical dilemmas, offer foundational insights that illuminate the complexities inherent in narrative research ethics. These researchers provide personal perspectives that help explore issues of rigor and trustworthiness (Nowell et al., 2017) while grasping the multidimensional nature of ethical challenges in qualitative research processes in narrative inquiry. Their experiences demonstrate how ethical considerations become dominant when investigating individual experiences through storytelling, where preserving participant autonomy and dignity requires continuous ethical reflection rather than one-time procedural compliance.

With all of the above perspectives, the ethical dilemmas commonly encountered in narrative research, including power dynamics, emotional vulnerability, and challenges to anonymity, are well-documented in the literature (Dhungana, 2022). However, these challenges are amplified in narrative inquiry, given its emphasis on authentic storytelling and deep relational engagement between researchers and participants. The intimate nature of narrative data collection, which often involves sharing personal stories and vulnerable experiences, creates a unique ethical terrain that requires sophisticated approaches to informed consent, confidentiality, and researcher-participant relationships. Universities and schools maintain rigorous research approval procedures to ensure quality standards and ethical considerations. Nevertheless, while critical for guiding researchers through complex situations from research inception to final reporting, these institutional frameworks may be inadequate to address the nuanced ethical challenges that emerge in the lived experience of narrative inquiry. The recurring ethical tensions in narrative research suggest a need for expanded ethical frameworks that integrate procedural requirements with relational ethics, cultural sensitivity, and philosophical grounding.

Whilst Western ethical principles provide essential foundations, the complex, contextual, and relational nature of narrative research may benefit from integration with broader Eastern wisdom traditions that emphasize holistic approaches to human dignity, continuous ethical reflection, and the cultivation of virtuous research practices. Eastern wisdom traditions, including Buddhist and Hindu philosophical frameworks, offer complementary perspectives on understanding ethical conduct that emphasize selfless action (i.e., *Karma*), righteous duty (i.e., *Dharma*), compassion (i.e., *Karuṇā*), and mindful detachment—principles that may provide valuable guidance for navigating the ethical complexities inherent in narrative research. Thus, this review addresses the critical need to understand ethical dilemmas in narrative research by synthesizing empirical evidence with philosophical insights from Eastern wisdom traditions while taking into account reflections from three experienced narrative researchers who grappled with them firsthand. Hence, the study aims to identify recurring ethical challenges in narrative research and explore solutions that integrate procedural ethics with relational, cultural, and philosophical approaches to ethical decision-making. Drawing on both Western academic literature and Eastern wisdom traditions, this review, informed by Buddhism and Hinduism as articulated in texts such as the Bhagavad Gita, aims to develop a more holistic framework for ethical practice in narrative inquiry that balances methodological rigor with human dignity.

## 2 Navigating ethics as qualitative researchers

As researchers, we typically understand ethics as a set of rules and values that determine what is morally right or wrong, providing a specific guideline for research activities that ensure the integrity of researchers (Dahal and Luitel, 2022; Poole, 2021). Initially, we might think that the ethical consideration of research is to be considerate toward research participants, thereby upholding ethical values that maintain societal wellbeing by ensuring research is credible and confidential. When we began our research, several concerns arose, and we realized that it is not just about maintaining credibility and confidentiality in the work but also about ensuring a rigorous practice of addressing enduring ethical dilemmas and challenges throughout the research process (Reid et al., 2018). For instance, many “wh” (what, how, and why) questions regarding ethical considerations arose in our minds, and we found that ethics, in general, is about doing the right thing in research (Reid et al., 2018). Later, we realized that it is not just knowing right from wrong as considerate researchers, but also having clarity and even greater confidence in disseminating our work to the public. Having said that, in our quest to understand the value of ethics in every research activity, we explored the Eastern wisdom tradition, considering that it emphasizes the value and consideration of people and moral behavior (Merriam-Webster, 2023). To deepen our understanding, we explored the linguistic historical origins of ethics, tracing its derivation. Ethics derives from the Greek word “ethos,” which means custom or character (Merriam-Webster, 2023). Thus, it typically evokes the goodness or wrongness of human lives, as discussed in the *Nicomachean Ethics* (Cooper, 1985). There is no single, absolute definition of “Ethics” because it is constantly evolving over time and in context. It is continuously related to socio-cultural and political contexts, so ethical standards are significant for protecting the authenticity of the subject matter and ensuring the validity of scientific exploration (Stutchbury and Fox, 2009).

Considering the above, ethics has always been used to distinguish right from wrong behavior, grounded in the principle of “no harm” to others. It often influences our actions and decisions due to different times, contexts, and personal beliefs. Research practices are directed by ethical guidelines grounded in philosophical principles—namely, promoting the wellbeing of others, doing good, and avoiding harm to others (Dahal and Luitel, 2022; Reid et al., 2018). No harm to anybody means respecting autonomy, where creativity is encouraged. However, in cases of conflict or when faced with two or more conflicting options, one may feel trapped or conflicted, which can hinder creativity in research and may also result in a lack of clear solutions (Poole, 2021). So, ethics is always considered an incredible philosophical research method that is crucial for knowing (Clandinin, 2007) whether the researchers have done justice or have gone through the complete procedure to ensure research participants' rights of wellbeing and dignity with respect (Stutchbury and Fox, 2009). Ethical dilemmas ensure that the participants are respected with dignity by maintaining self-integrity and credibility throughout the research process. While discussing ethics as an ethical consideration in research, it is related to understanding the credibility and confidentiality of research (Ginting, 2022). Thus, ethics always ensures the application of moral principles and guidelines for conducting scientific research involving human participants, other living beings, or any subject matter (Steffen, 2016).

Therefore, the ethical consideration involves a principle aimed at minimizing potential harm to participants and researchers. These principles consistently emphasize honesty, fairness, and integrity toward oneself and others (Dahal and Luitel, 2022). Such studies helped researchers deepen their understanding of the duties and responsibilities of research work, particularly regarding research credibility and confidentiality (Ginting, 2022). Since ethics has been a crucial guiding principle for researchers, who are always aware of how to make their research credible in front of others. In doing so, we navigate the dialectical dilemmas (e.g., ethical challenges) in various contexts to make our research morally acceptable to the broader community. Keeping all these in mind, we questioned our general understanding of ethics. We examined ourselves as researchers and attempted to explore the actions (i.e., Karma) that we encountered during our research journeys. To this end, we began by exploring ethics and its dilemmas from Western to Eastern wisdom traditions and systematically reviewed 16 empirical studies (2014–2023). To explore ethics in greater depth, we examined Eastern philosophical traditions (Hindu and Buddhist traditions). We uncovered insights into the role of ethics in researchers' lives, particularly in fostering credibility and integrity within the research process.

## 3 Understanding ethics through the lens of Eastern perspectives

Ethics always evokes the act without expectation, which means *Karma* without expectation of results and/or outcomes (Prabhupada, 1986). In other words, ethics refers to the rightness and wrongness of human behavior, as well as the consequences of one's actions, and is known as *Karma* in Eastern philosophy. It is always value-laden and rooted in traditions and philosophy, which makes it different from Western values. Some Eastern values in understanding ethics related to moral virtues (Dahal and Luitel, 2022) help prevent one from becoming immoral to others. In this regard, the different Eastern philosophies are ingrained in our daily activities, making us aware that we should perform any action (i.e., *Karma*) morally. Nevertheless, it is more contextual, as it differs from country to country, context to context, and even in South Asia and Nepal.

Similarly, we found Chinese philosophy “Confucianism,” which emphasizes moral virtues and living harmoniously with others and is known for its value of *Ren*. It primarily focuses on humanity, humanness, and benevolence (Sun, 2013), which entails treating people with kindness and empathy. There, we found “Taoist ethics,” which means living in accordance with the natural way of life, where Tao is understood as a natural path in Chinese religion (Mark, 2016). Everything needs to be aligned with the flow of nature so that things can be harmonious and make the world a better place to live in. Living life with oneself, others, and the world of changes is called leading a virtuous life as a whole. Likewise, Buddhism holds that ethics reduces pain and suffering, leading to enlightenment (*Nirvana*), the foundation of ethics (Goodman, 2021). These beliefs help guide people in living a moral and compassionate life without harming others. The study by Goodman (2021) revealed that ethics in Buddhism is akin to a moral discipline in life, involving a commitment to follow specific moral guidelines to foster trust in others. Trust is one of the most important tools for convincing research participants to participate, which is significantly important for researchers. Therefore, trust and rapport-building are essential elements for conducting research smoothly during the data collection process.

We have primarily discussed ethics and its significance in research; however, we aimed to introduce the concepts of selflessness and commitment to work (i.e., *Karma in Sanskrit*). However, science has brought a tremendous transformation in the name of technological and economic development. People have been on the safer side so far, but morally and spiritually, they have been away (Patra, 2020). In this materialistic world, when conducting research, one must be guided by moral values and possess virtues in every intention of the research work (Karma as a researcher). According to Hindu ethics, *Dharma* (virtue) refers to the moral duties on the path to righteousness. Aligning with one's own *Dharma* comes only from rigorous *Niskama karma (selfless action) (Patra*, *2020**), which brings* an unbiased reality to society. Lord Krishna in the *Bhagavad Gita* conveys the wisdom of ethical living and detachment through *Karma Yoga* (Prabhupada, 1986), which refers to ethical duties and behavior of being (people). Since *Karma* is always related to *Dharma*, and one should move ahead without being attached to success or failure, non-attachment is a key concept in *Karma Yoga*, as outlined in the *Bhagavad Gita*. The essence of *Vedic* wisdom that comes from the *Gita*, which always encourages one to perform one's own duty, is not less than the right attitude and is not less than the substantial path of self-contentment. The *Bhagavad Gita*, commonly known as the Gita, is a revered Hindu scripture. It constitutes a key section of the epic Mahabharata. It presents a synthesis of Indian spiritual philosophies—integrating the Vedic principle of *Dharma*, the analytical paths of Jnana yoga (i.e., knowledge), and the devotional approach of Bhakti Yoga. Service and compassion toward duty are not just an external force that continues the journey, but also a purification of the mind, aligning with the inner purification of thoughts and intentions. *Karma Yoga* encourages one to live with integrity, selflessness, and righteousness while performing research duties and responsibilities. The study by Patra (2020) suggests that moral values in everyone's life significantly impact “professional, personal and societal development that makes life easier and happier” (p. 1026). Patra (2020) further emphasizes the holistic impact of moral values, suggesting that they serve as a foundation for both individual and collective wellbeing, thereby fostering positive relationships.

Since a good life is related to happiness, as Aristotle (1985, as cited in Spratt, 2017) stated, this philosophy raises questions about how people strive to achieve it. However, it is difficult to find a single definition of ethics as it is discussed in various ways above. Suppose it is always related to human action [i.e., behavior(s)], which is ever-changing over time and context. How can we establish a reliable model to maintain ethics and its dilemmas so that the complexity of human actions does not harm any research? Likewise, Othman and Abdul Hamid's (2018) study highlighted that sharing ethical values between two individuals is crucial during research to minimize any harm caused by human behavior. However, some ethical dilemmas arise in specific research contexts, adding complexity to the research. Therefore, navigating ethics from West to East has led to an understanding of its dynamic aspects and ever-changing complexity. As researchers, one must study ethics from the West to the East, as the world revolves around its philosophical foundations. To serve the purpose of this article, we have formulated two research questions to explore the ethical dilemmas that researchers faced and perceived during research, in general, and specifically in narrative research.

a. How do researchers navigate ethical dilemmas during the narrative research process?
b. What are the perceived ethical challenges reported by researchers in narrative research?

## 4 Materials and methods

This article conducted a review aimed at developing knowledge synthesis of current literature on ethical concerns of qualitative research with a particular focus on narrative research, informed by Eastern wisdom traditions (i.e., Hindu and Buddhist philosophy) and the lived experience of three university faculty members who have employed narrative inquiry methodology in their graduate-level research and ongoing academic practice. The methods applied to review principles to reduce the risk of bias and increase the validity of results (Hansen et al., 2021) include a multistage process of identification, screening, eligibility assessment, and inclusion.

### 4.1 Search strategy and data sources

The search in this review is limited to references published between 2014 and 2023. The search strategy favored articles with terms such as “ethics,” “ethical dilemmas,” “ethics in qualitative research,” and “narrative research or narrative inquiry.” The review included peer-reviewed journal articles and pertinent methodological book chapters that generally offered commentary on ethical issues in qualitative methodologies, particularly narrative inquiry and perspectives rooted in Eastern philosophy (Hindu and Buddhist traditions). The studies were drawn from various global contexts to ensure geographical diversity, including Iran, the United Kingdom, Hong Kong, Bangladesh, Canada, Malaysia, South Africa, the United States, China, Nepal, Indonesia, Brazil, and Costa Rica.

### 4.2 Review selection process

**Figure 1:**
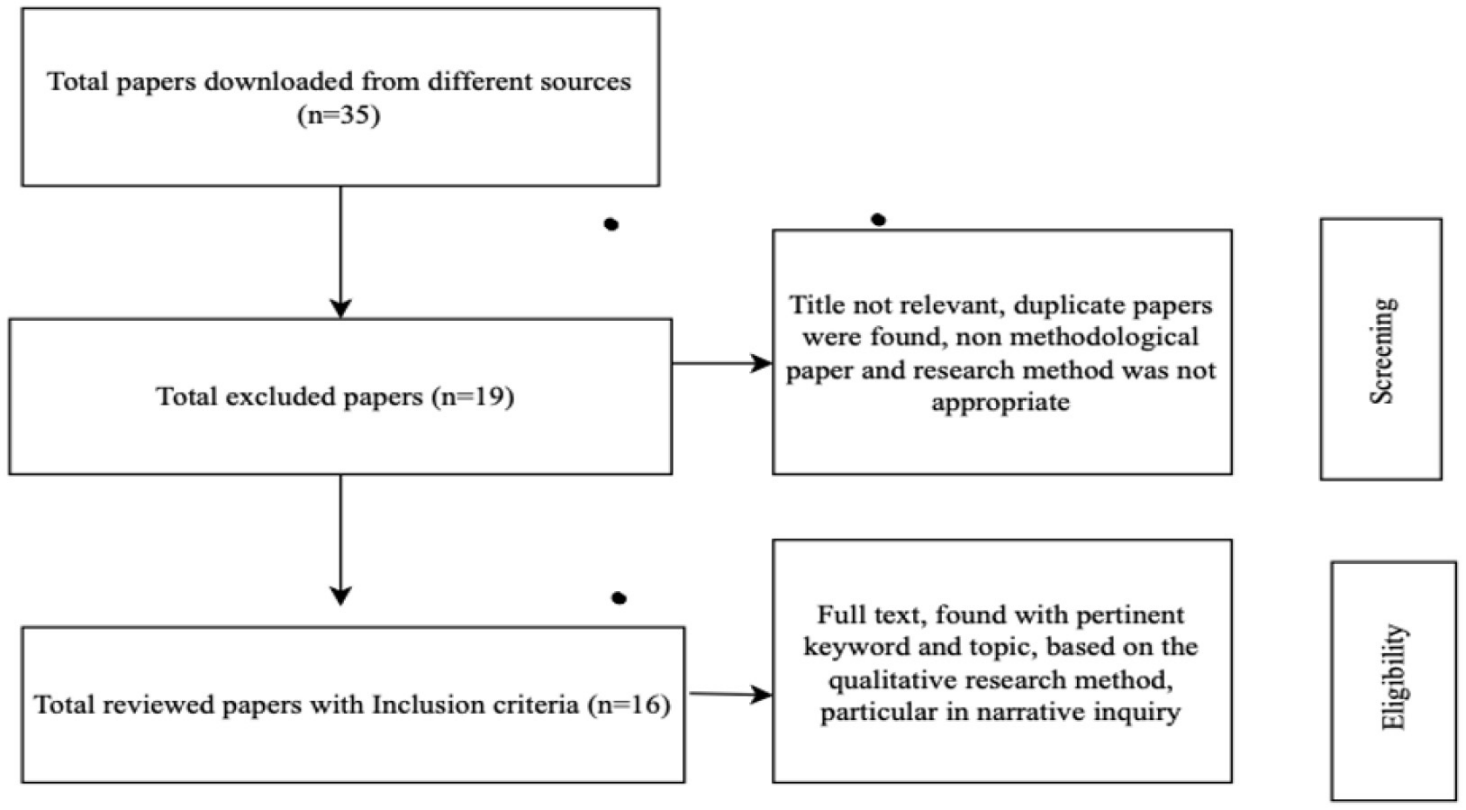
Flowchart to meet the inclusive criteria of the review process.

The selection procedure followed a multistage scheme, as outlined in the flowchart (Figure 1). The first phase, a screening of titles and abstracts, involved conducting a preliminary search through the databases to compile a list of articles for further review. In this screening process, abstracts and titles were examined for connections to the two central themes—ethics and qualitative research. During the eligibility stage, full texts of potentially relevant articles were assessed against predefined inclusion criteria, which included a focus on ethical dilemmas or challenges in qualitative research methodology, inclusion of empirical research, theoretical discussions, or critical literature reviews, publication in English within the 2014–2023 time frame, and explicit discussion pertinent to the research questions—particularly those related to narrative inquiry. Finally, in the inclusion phase, articles that met all eligibility criteria were selected for synthesis, while those deemed irrelevant to the specific focus on ethical dilemmas in qualitative or narrative research were excluded. From an initial pool of 35 downloaded articles, 16 were selected for in-depth review and content analysis based on thematic relevance and scope.

### 4.3 Data extraction and synthesis

A qualitative content analysis approach was employed to examine the selected literature, following the systematic procedures outlined by Krippendorff (2013). It involved coding and categorizing key themes, ideas, and structures related to ethical decisions. The meta-analysis centered on the typologies and the descriptions of dilemmas faced, the contexts within which the dilemmas arose (e.g., in relation to particular qualitative methods or data collection points), potential strategies or issues presented to address the dilemmas, and the difficulties that were found to be particular or strongly characteristic of narrative work. Furthermore, the researchers systematically extracted and reported the major attributes of the 16 included studies—such as the author(s), the year the research was conducted, the study context, and the focus area—in a tabular form (see Table 1). The synthesis used a meta-synthesis approach, combining study findings to construct a more comprehensive picture of the ethical terrain. This method was employed to identify influential variables and common patterns, in accordance with the working mechanisms of qualitative meta-synthesis, as explained by Hansen et al. (2021). Table 1 illustrates the list of ethical dilemma studies.

**Table 1:** The study of ethical dilemmas in qualitative research from different contexts worldwide.

**S.N**.	**Author(s) and year**	**Context**	**Area of concentration**
1	Sanjari et al. (2014)	Iran	Ethical challenges of researchers in qualitative studies
2	Gillian (2017)	UK	Narrative inquiry and the problem of representation: giving voice, making meaning
3	Yung (2016)	Hong Kong	Ethical dilemmas in shadow education research: a qualitative study
4	Hultgren et al. (2016)	Bangladesh	Ethics in language and identity research
5	Steffen (2016)	UK	Ethical considerations in qualitative research
6	Park et al. (2016)	Canada	Ethical tensions as educative spaces in narrative inquiry
7	Nowell et al. (2017)	Canada	Thematic analysis: striving to meet the trustworthiness criteria
8	(Othman and Abdul Hamid 2018)	Malaysia	Dealing with un (expected)ethical dilemma: Experience from the field
9	Ngozwana (2017)	South Africa	Ethical dilemmas in qualitative research methodology
10	De Costa et al. (2021)	USA	Navigating ethical challenges in Second language narrative inquiry research
11	Poole (2021)	China	Narrative inquiry and relational ethics: negotiating the lived experiences of international schoolteachers in China
12	Dahal and Luitel (2022)	Nepal	Understanding and encountering the ethics of self and others in autoethnography
13	Ginting (2022)	Indonesia	Ethical research dilemmas and their implication in English language teaching studies
14	Taquette and Borges da Matta Souza (2022)	Brazil	Ethical dilemmas in qualitative research: a critical literature review
15	Charpentier-Jimenez (2023)	Costa Rica	Students' perception of ethics in applied linguistic research
16	Laryeafio and Ogbewe (2023)	UK	Ethical consideration dilemma: systematic review of ethics in qualitative data collection

### 4.4 Supplementary data collection

To augment the literature review and situate the findings in the lives of researchers and three experienced university faculty members who have employed narrative inquiry methodology in their graduate-level research and ongoing academic practice, a qualitative survey was used. A structured qualitative interview protocol was applied. Through an open-ended interview, participants were invited to articulate their experiences and attitudes toward ethical dilemmas in conducting narrative research. The views of three experienced university faculty members who have employed narrative inquiry methodology in their graduate-level research and ongoing academic practice were included in the data and used to inform the “perceived ethical dilemmas” that emerged (see Table 2). Their contribution added to the conversation by revealing problems and bringing a real-world dimension to the theoretical outcomes and/or results.

**Table 2:** Identified ethical dilemmas discussed in narrative research from different contexts and participants' experiences.

**Ethical dilemmas**	**Contexts**
Method	•Not clear with the methodology of the research •Due to the lack of resources used in the field
Informed consent	•Sometimes, due to a lack of clear information, participants may not agree •Not explaining the real purpose of the research may create chaos
Confidentiality and anonymity	•Not providing meaningful narratives to balance the confidentiality of the participant •Failure to delete participants' profile details
Lack of privacy	•Due to a lack of record-keeping in your drive •Sharing stories with other peers about the data collection experience •Sharing raw data with supervisors during research discussion
Power dynamic	•Not able to balance the power between the researcher and participants •Not creating an open and friendly environment for participant for their storytelling
Lack of privacy	•Due to a lack of record-keeping in your drive •Sharing stories with other peers about the data collection experience •Sharing raw data with supervisors during research discussion
Power dynamic	•Not able to balance the power between the researcher and participants •Not creating an open and friendly environment for participant for their storytelling
Respect or no harm	•Not treating participants as yourself •Not offering any monetary things or goods for the interview
Cultural sensitivity	•Due to a lack of respect, participants may not be interested in taking part in the interview multiple times •Not being mindful during the emotional sharing of participant stories •Not having a cultural understanding of its norms and values may influence participants' willingness to share
Vulnerability (emotional impact)	•Being a researcher, putting yourself too much into a participant's emotions may create dilemmas •Emotionally draining, along with participants, may harm your research •Recognizing and mitigating the emotional toll on participants who revisit traumatic or sensitive experiences during storytelling

### 4.5 Analysis procedures

Sixteen studies were analyzed (2014–2023), alongside insights from Eastern wisdom traditions (Hindu and Buddhist). A particular sub-analysis was conducted to detail ethical issues in qualitative research, in general, and in narrative research across different contexts, and to include three experienced university faculty members, as mentioned above, who have employed narrative inquiry methodology in their graduate-level research and ongoing academic practice. Eight articles were found to focus on ethics in the application of qualitative methods. Of these, four are empirical works—Gillian (2017), Park et al. (2016), De Costa et al. (2021), and Poole (2021)—that addressed ethical considerations within the narrative research framework. The other four included studies—Sanjari et al. (2014), Yung (2016), (Steffen (2016), and Taquette and Borges da Matta Souza (2022)—raised the predominantly relevant issue of ethical challenges in qualitative research more broadly, providing crucial contextual understanding. This analysis was limited to the methodological features exclusive to narrative inquiry, including the use of lived stories, the development of researcher-participant relationships, and issues of power and emotionalism. These were analyzed to identify how they may lead to or give shape to particular ethical quandaries in narrative research.

## 5 Findings

**Figure 2:**
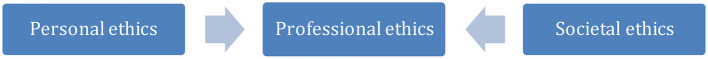
Relational analysis of ethics.

The synthesis results are narratively described, visually summarized, and tabularly summarized to enhance clarity and transparency. Table 1 summarizes the included studies and provides an overview of context and focus. A summary of the core ethical dilemmas identified, their categorization, and examples from the literature and participant responses is presented in Table 2. A flowchart (Figure 1) has been created to illustrate the selection process, encompassing the identification, screening, eligibility, and inclusion phases. Figure 2 provides a conceptual framework that describes the relational aspects of ethics (i.e., the ethics of relation), including ethics toward participants, ethics of self-care, and ethics toward society or readers. Table 3 illustrates Eastern philosophy solutions to ethical dilemmas in narrative research. This methodical process provided a thorough, evidence-based illustration of ethical dilemmas in qualitative research, with a particular focus on narrative research and/or inquiry.

**Table 3:** Eastern wisdom tradition and solutions to ethical dilemmas in narrative research.

**Ethical dilemma**	**Eastern concept**	**Philosophical foundation**	**Practical application**	**Supporting literature**
Power dynamics	Karma Yoga (selfless action)	Acting without attachment to outcomes or personal gain	Researchers approache participants as equals, focusing on service rather than data extraction; eliminate coercive dynamics through genuine selflessness	Prabhupada, 1986; Patra, 2020
Informed consent	Dharma (righteous duty)	Moral obligation to uphold truth and justice in all actions	Ongoing, co-constructed consent process that honors participant autonomy as a sacred duty; continuous dialogue about participation	Patra, 2020; Dahal and Luitel, 2022
Emotional vulnerability	Karuṇā (compassion) + Upekkha (equanimity)	Boundless compassion balanced with mindful detachment	Researchers maintain an empathetic presence while avoiding emotional enmeshment and support participants without absorbing their pain	Goodman, 2021; Buddhist teachings
Anonymity and confidentiality	Ahimsa (non-harm)	Fundamental principle of causing no harm to any being	Protecting participant identity becomes a moral imperative; creative anonymization that preserves dignity while maintaining narrative authenticity	Hindu/Buddhist ethics; Taquette and Borges da Matta Souza, 2022
Cultural sensitivity	Ren (benevolence/humanity)	Confucian virtue emphasizing human-heartedness and respect	Genuine engagement with participants' cultural contexts; the researcher cultivates deep respect for diverse worldviews and practices	Sun, 2013; Confucian ethics
Trust building	Saddha (faith/trust) + Metta (loving-kindness)	Buddhist concepts of cultivating mutual trust through loving-kindness	Researchers develop authentic relationships through consistent loving-kindness; trust emerges naturally from genuine care	Buddhist psychology; Goodman, 2021
Researcher reflexivity	Svadhyaya (self-study)	Yogic practice of continuous self-examination and learning	Ongoing introspection about motivations, biases, and impact; the researcher as a reflective practitioner committed to growth	Yogic philosophy; Dahal and Luitel, 2022
Reciprocity and benefit	Dana (generous giving)	Buddhist/Hindu principle of selfless giving without expectation	Research designed to genuinely benefit participants and communities; knowledge sharing that serves collective well-being	Buddhist/Hindu ethics; Patra, 2020
Narrative authenticity	Satya (truthfulness)	Commitment to truth in thought, word, and deed	Faithful representation of participant narratives; researcher commitment to authentic storytelling that honors lived experience	Hindu ethics; Gillian, 2017
Emotional boundaries	Vairagya (detachment)	The yogic principle of non-attachment while remaining fully engaged	Researchers maintain professional boundaries through spiritual detachment; being present but not possessive of participant stories	Bhagavad Gita; Prabhupada, 1986
Contextual sensitivity	Wu Wei (effortless action)	Taoist principle of acting in harmony with natural flow	Researchers adapt ethical approaches to fit cultural and situational contexts; flexible responsiveness rather than rigid rule-following	Taoist philosophy; Mark, 2016
Collective responsibility	Sangha (community)	Buddhist concept of interconnected community responsibility	Research conducted with awareness of broader community impact; ethical decisions consider collective wellbeing	Buddhist community ethics; Goodman, 2021

### 5.1 Ethical challenges and considerations in narrative research

In this section, we have selected eight articles (*n* = 8) to review based on qualitative research and only four articles (Gillian, 2017; Park et al., 2016; De Costa et al., 2021; Poole, 2021) out of eight empirical research articles, which have discussed the ethical issues that are found in narrative research. Several studies (Sanjari et al., 2014; Yung, 2016; Taquette and Borges da Matta Souza, 2022) have discussed ethical dilemmas in qualitative research. People are well-informed about primary qualitative research approaches, such as narrative inquiry, case study, ethnography, action research, phenomenology, and grounded theory (Creswell, 2007). Additionally, they are well-versed in qualitative research processes, including the roles of researchers involved. However, when researchers lack a balance between themselves and participants due to unnecessary involvement in participants' emotions, it may create dilemmas in research (Sanjari et al., 2014). In almost all qualitative research, the primary data collection techniques are the same: observation, interview, open-ended questions, verbal reports, reflexivity, and discourse analysis (Butina, 2013). However, some essences and qualities vary from one research study to another. For instance, when discussing narrative inquiry as a form of qualitative research, we often consider research where researchers carefully and thoughtfully produce in-depth information about participants from their told stories and lived experiences, thereby generating a realistic picture of the real world (Heigham and Croker, 2009). Sometimes, power dynamics can create dilemmas if a narrative inquirer fails to create a supportive environment for participants to share their stories (Yung, 2016). A narrative inquiry is a research methodology within the qualitative research approach. It is viewed as a mode of processing human personal experience for meaning-making, and their experiences are illustrated through their told stories (Webster and Mertova, 2007). As discussed above, being a narrative inquirer can pose dilemmas in research if too much emphasis is placed on a participant's emotions (De Costa et al., 2021). Therefore, the researcher's emotions and vulnerability might interfere with data collection if the researcher becomes overly involved in participants' personal experiences or emotions, which should be acknowledged and well-balanced.

In narrative inquiry, researchers collect data from lived experiences and told stories (Barkhuizen et al., 2014) through trust and a strong rapport with research participants (De Costa et al., 2021). So, it involves a series of events based on an individual's told stories and lived experiences, which are eventually employed to disseminate newly generated knowledge to the outside world in an ethical manner. Our focus centered on ethics in qualitative research, particularly within the context of narrative inquiry. As both language teachers and researchers, we experienced a sense of vulnerability during data collection, especially when sharing similar emotions with participants. This shared professional background can lead to a mutual understanding of challenges and uncertainties, which, according to De Costa et al. (2021), may contribute to a shared sense of vulnerability regarding career prospects and professional dilemmas. Such dynamics can create ethical complexities for the researcher. Sharing the stories can be emotionally draining, which may damage research; therefore, it is essential to be aware of one's emotional state during the research process (Yung, 2016).

Narrative inquiry offers rich and valuable insights through meaning-making (Gillian, 2017); however, while generating such insights, a researcher must navigate some ethical dilemmas. However, it is the researcher's responsibility to share authentic findings as an accurate representation of the research, conveying a significant message to the world (Gillian, 2017). Therefore, researchers must integrate ethics throughout the entire research process to build trust and foster a good relationship, which helps generate reliable data. Moreover, a good relationship between the researcher and participants enables both to overcome ethical dilemmas encountered during research (Poole, 2021). According to Poole (2021), narrative inquiry researchers must possess the ability to comprehend complex issues, such as ontology, epistemology, and axiology, in order to balance between the two parties (participants and researcher). Establishing a good relationship with the research participant is considered one of the key components for balancing power dynamic issues and ensuring the quality of research in general. It also provides more accurate information and helps avoid challenges that can create a friendly environment for participants (Yung, 2016). Therefore, qualitative research is considered a research approach rather than a specific technique (Morgan and Smircich, 1980), which is inherently aligned with the nature of the social sciences in understanding its phenomena.

Ethical considerations in qualitative research are required at every stage of data collection, analysis, and the overall research process, yet they are often overlooked. When a researcher feels superior to a participant and fails to treat them as an individual, it also creates ethical dilemmas (Laryeafio and Ogbewe, 2023). It should be viewed as an agency filled with dynamic knowledge. According to Heigham and Croker (2009), qualitative research is conducted in natural settings, and every available resource is used as data to interpret phenomena in a meaning-making context. The study by Park et al. (2016) emphasizes the importance of understanding who we are and what we are becoming in relation to participants as we work together in a real-world context. This is the nature of qualitative research, which generates a realistic picture through thoughtfully designed activities that respect research participants (Heigham and Croker, 2009).

Adopting a qualitative approach is worthwhile for understanding human characters, as it relates to subjective value, which is why it is value-laden. The issue is purely person-centered. So, maintaining anonymity and confidentiality (Ngozwana, 2017) in research is inevitably essential. As researchers, it is also appropriate to adopt qualitative research procedures (Ginting, 2022). In the words of (Taquette and Borges da Matta Souza 2022), respect for others is one of the ethical principles that highlights the value of every individual throughout the entire research process. Disregard of autonomy may damage anyone involved in the research committee (Taquette and Borges da Matta Souza, 2022). The literature reports that rigorous work, continuous consent, understanding the issues of ontology, epistemology, and axiology in research, and cultivating interpersonal rapport are key instruments for overcoming ethical dilemmas.

### 5.2 Ethical tensions in narrative research: balancing research with participants' rights

We reviewed literature from several studies to explore the ethics of narrative research, and some evolutions have been identified. While conducting narrative research, we collected and analyzed participants' narratives to gain a deeper understanding of the meanings and insights they offered about their ethical dilemmas. Since the preliminary stages of narrative inquiry, such as conducting interviews and collecting stories (Clandinin, 2007), ethical considerations play a crucial role from the beginning of the research. However, the approach raises moral issues; for instance, participants may reveal private or potentially sensitive information, which necessitates that researchers maintain confidentiality, obtain informed consent, and ensure participants' wellbeing at all stages of the study. In many universities, researchers lack a clear ethical framework, leading to a lack of established guidelines to ensure the ethical quality of research (Charpentier-Jimenez, 2023).

(Othman and Abdul Hamid 2018) shared their story of encountering ethical dilemmas while researching a sensitive issue in the field. For researchers, preserving participants' integrity by maintaining anonymity and confidentiality is challenging throughout the research process, particularly in qualitative and narrative research (Othman and Abdul Hamid, 2018). Ethical considerations in narrative inquiry often revolve around balancing the researchers' objectives with participants' autonomy. They face challenges throughout the research procedures due to the demanding nature of the research (Ginting, 2022). According to Ginting, demanding impacts include low self-esteem, loss of autonomy, and strained social relations, which can increase ethical conflicts during research. However, real-world challenges often require researchers to go beyond formal protocols and engage in reflexive, context-sensitive ethical decision-making. Sometimes, dilemmas emerge that require continuous consent to balance their benefits and costs (Ginting, 2022). Nowell et al. (2017) reported that continuing to obtain consent helps build research trustworthiness. It addresses challenges such as informed consent, anonymity, and confidentiality, which are the major dilemmas encountered during research (Ngozwana, 2017). It has been found that challenges arise when a researcher fails to consider their duties during the research and becomes fully immersed in the role, treating others as human beings (Park et al., 2016). Approaching research with empathy and recognizing participants as human beings first enables us to connect with their emotions, ensuring that their involvement is both voluntary and respectful (Ginting, 2022). Therefore, threats are always considered unethical and must be avoided to ensure participants' privacy and prevent them from being intimidated by anything related to the research. The consent form serves as evidence of ethical considerations in the research. Without the participant's consent, no one can persuade them to participate in their research.

Participants' consent should include some clauses. If a participant is unwilling to continue after a few rounds of interviews, we cannot take action against them, nor can we reduce their self-esteem (Taquette and Borges da Matta Souza, 2022). Persuasion in research is regarded as unethical, particularly when it involves offering monetary incentives to influence participation. Researchers must respect participants' autonomy, including their right to withdraw from a study at any time, as Laryeafio and Ogbewe (2023) emphasize that clearly communicating the voluntary nature of participation and the freedom to exit whenever discomfort arises fosters trust between researchers and participants, making individuals feel safe and respected during the research process. Similarly, (Charpentier-Jimenez 2023) features the ethical challenges that arise when participants are deceived or subjected to prolonged interviews, highlighting the importance of transparency and consideration in research design. Moreover, this activity minimizes the risk of harm to research participants.

In some cases, researchers are theoretically informed about ethics but are practically unable to maintain it in the field (Dahal and Luitel, 2022). According to Dahal and Luitel (2022), three domains of ethics in qualitative study, “ethical toward people who are participating in the research, second, ethics of self-care, even critically reflexive about one's own work, and third, being ethical to the society/for our readers” (p. 2681) are prominent in research. They discussed relational ethics, which is inherently intertwined (Poole, 2021), and highlighted the importance of ethical understanding in research. Maintaining our ethics aligns with professional ethics, resulting in good outcomes without harming others. Whatever we gather from our research, we tend to disseminate to the world and to readers in our academic community. Our personal belief in not harming others upholds societal ethics as well. Figure 2 illustrates the relational analysis of ethics.

We have tabulated some of the identified dilemmas from the narrative review of empirical research (*n* = 16) and the narratives of three narrative researchers. These challenges highlighted the need for a nuanced approach to ethical decisions that extend beyond university and other organizational requirements. In the following discussion, we present a solution that addresses ethical dilemmas, drawing on the findings of this review and insights from Eastern philosophy, to benefit all qualitative researchers, including those engaged in narrative research, in reducing ethical dilemmas throughout the research process. Table 2 identifies ethical dilemmas discussed in narrative research from various contexts and participants' experiences. Table 3 illustrates the Eastern philosophy solutions to ethical dilemmas.

The key findings from this review reveal recurring ethical challenges, including continuous informed consent, power dynamics, privacy (including confidentiality), emotional impact, and the vulnerability of both parties (i.e., the researcher and the participant), which are some of the perceived ethical dilemmas. This article examines Eastern philosophy's approaches to addressing ethical dilemmas in narrative research, encompassing power dynamics, informed consent, emotional vulnerability, anonymity and confidentiality, cultural sensitivity, trust-building, researcher reflexivity, reciprocity and benefit, narrative authenticity, emotional boundaries, contextual sensitivity, and collective responsibility.

## 6 Discussion

Narrative research offers insight through lived stories and demands careful ethical navigation as researchers face tensions between methodological rigor and participants' rights. So, involvement creates power dynamics that risk suppressing voices or exploiting vulnerability if not addressed by rapport (Yung, 2016; De Costa et al., 2021). Emotional risks affect participants and researchers—particularly those who share cultural or professional contexts—with possible distress, over-identification, and compromised objectivity (De Costa et al., 2021; Yung, 2016; Gillian, 2017). Maintaining anonymity presents a significant challenge in detailed narratives, where even subtle identifiers or the manner in which data is processed can inadvertently reveal a participant's identity (Othman and Abdul Hamid, 2018; Ngozwana, 2017). Consent should be viewed as an ongoing and dynamic process, allowing individuals to withdraw when sharing becomes emotionally burdensome (Ginting, 2022; Taquette and Borges da Matta Souza, 2022; Nowell et al., 2017). Ethical narrative research is inherently relational, emphasizing respect, cultural sensitivity, and minimizing harm while highlighting the mutual responsibilities of the researcher and the researched (Poole, 2021; Dahal and Luitel, 2022; Taquette and Borges da Matta Souza, 2022). Reflexivity, contextual awareness, and a deep commitment to ethical responsibility that extends beyond formal guidelines are essential for preserving trustworthiness and integrity throughout the narrative research process (Poole, 2021; Dahal and Luitel, 2022). The lived experiences of R1, R2, and R3 exemplify these ethical complexities in R1's struggle with participant trust demonstrates how inadequate communication compromises both ethical relationships and data quality. R2's challenge in maintaining anonymity while preserving narrative authenticity highlights the fundamental tension between protection and representation that is unique to narrative inquiry. R3's reflection on managing theoretical frameworks while maintaining relational responsibilities highlights the cognitive burden that ethical narrative research imposes on researchers. These experiences illustrate what we term “reciprocal vulnerability”—where both parties are exposed to emotional risk through sharing and receiving personal narratives.

Thus, narrative research offers insights through lived experiences, but it also presents ethical challenges that extend beyond standard procedural ethics. These challenges—such as power dynamics, emotional vulnerability, and anonymity—are intrinsic to the storytelling process and demand a more relational and reflexive approach. To address this, we propose an ethical framework grounded in Eastern wisdom traditions that foregrounds selfless action *(Karma Yoga*), righteous duty (*Dharma*), compassion (*Karuṇā*), and mindful detachment. *Karma Yoga* encourages researchers to engage with participants not to extract data but to fulfill the duty of understanding with integrity, thereby reducing coercive dynamics and fostering trust (Prabhupada, 1986; Patra, 2020). Dharma reframes ethical responsibilities, such as consent and anonymity, as ongoing, co-constructed duties rather than one-time formalities (Patra, 2020; Dahal and Luitel, 2022). Compassionate detachment, derived from Karuṇā and teachings in the Bhagavad Gita, enables researchers to strike a balance between empathy and clarity, thereby supporting participants without experiencing emotional exhaustion (Goodman, 2021; Prabhupada, 1986). Cultural sensitivity is addressed through Ren (benevolence), which calls for genuine relational engagement and respect for participants' values (Sun, 2013). Hence, these principles transform ethical tensions into opportunities for personal and professional growth, positioning the researcher as a reflexive practitioner guided by selflessness, duty, compassion, and mindfulness. When integrated with Western procedural ethics, this Eastern-informed framework offers a more holistic and humane foundation for qualitative research in general and narrative research in particular.

This integration suggests practical applications, including ongoing consent dialogues rather than one-time forms, collaborative anonymization in which participants help determine the appropriate identifying details, and mutual acknowledgment of vulnerability, creating frameworks for shared support. The framework addresses systemic challenges noted by (Charpentier-Jimenez 2023) regarding inadequate institutional ethical guidance for qualitative research by proposing a philosophical transformation alongside policy modification. Therefore, this review brings together empirical studies from 2014 to 2023 that suggest, considering the vast array of added empirical evidence and Eastern philosophical wisdom and tradition connected to multiple dimensions of confidentiality, that characterize both the problems and promises intrinsically to narrative research and/or inquiry. The standard ethical guidelines typically address issues such as informed consent, anonymity, power dynamics, emotional disclosure, and cultural sensitivity. The dilemma is that narrative research requires deep relational engagement, with ethical integrity linked to procedural processes (of reflexivity, relational ethics, and trust-building). Drawing on Hindu *Karma Yoga* and Buddhist compassion, the review presents an ethics-grounded conceptual framework centered on the balance between detachment and attachment, duty-practice, and mutual cleansing. It recommends context-specific processes, such as ongoing consent negotiations and co-constructed techniques for maintaining anonymity, as well as mutual consideration of vulnerability, to reduce power dynamics. Thereby, it asks scholars to dance with ethical tensions as sites of transformation and to couple Eastern and Western orienting frameworks, rendering ethical dilemmas not as barriers but as possibilities for richer integrity and co-constructed meaning-making.

This perspective transforms ethical challenges from obstacles into “educative spaces” (Park et al., 2016) that deepen understanding of research relationships and ethical development. The goal is not to eliminate ethical dilemmas inherent to narrative research but to transform them into opportunities for deeper ethical understanding, more authentic research relationships, and meaningful contributions to knowledge and social transformation.

## 7 Conclusion

Ethics is central to narrative research and inquiry, influencing trustworthiness and the persuasiveness of study findings. Researchers from all fields must navigate every stage involving ethical considerations to ensure the rights and well-being of individuals participating in their studies. It is also crucial to acknowledge the rights of those whose specimens and data are used in research, since their human involvement is inherent to the research process. The fundamental ethical principles of respect for research participants and protection of privacy are evident. This review examines ethical paradoxes and aspects of the research process from both Western and Eastern perspectives, contributing to a more complex understanding of the issues that confront narrative-oriented researchers. Discrepancies and misunderstandings of ethical guidelines might complicate the situation, leading to repeated ethical dilemmas throughout the research process. Identifying and preempting ethical issues is essential to ensure quality in any research project. Before conducting interviews or collecting data, interviewers should be well-versed in consent forms, interview records, and information to emphasize the importance of informed and voluntary participation. From the review, reflective of the agreement, the fundamental ethical issues in qualitative studies encompass informed consent, anonymity, privacy, voluntarism, the right to withdraw, a conducive and friendly space, and confidentiality, which are highly valued. As a result, any infringements or lack of understanding of ethical values give rise to ethical issues and the possibility that research may lose its course entirely. To further enhance the ethical quality of research, researchers should give greater attention to established, well-defined, and discipline-specific ethical guidelines. Training and workshops on ethical conduct can help researchers identify and proactively manage ethical challenges.

Additionally, fostering a collaborative culture among researchers who share experiences will enhance their ability to tackle ethical dilemmas in narrative research, particularly in qualitative studies. Regularly involving ethical audits and reviews throughout the research process may ensure commitment to ethical standards, protect participants' rights, and enhance the trustworthiness of research findings. Thus, educational institutions and grant agencies should promote ethics as a foundation for the responsible conduct of research in any form.

## 8 Implications and limitations

This review features the importance of reflexive, relationship-based ethical frameworks for narrative work, grounded in the need to respect participants' dignity and maintain the researcher's integrity. Drawing from the Eastern wisdom traditions—*Karma Yoga* (selfless action) and *Dharma* (right duty)—the article suggests that ethical discipline be built on foundations that engender trust and respect, transcending procedural legalism. The procedures in which power dynamics, emotional vulnerability, and cultural sensitivity intersect and give rise to dilemmas are discussed, and the implications for future practice are considered, including the development of discipline-specific ethical guidelines for qualitative researchers, reflexivity, and the cultivation of learning communities. Institutionalizing these measures would establish the dialectic of methodological rigor and ethical responsiveness as a hallmark of research and/or inquiry. The review is limited by its methodological and contextual boundaries. The review identified only 16 original studies (2014–2023), potentially missing more subtle ethical issues in underrepresented settings or in non-English-language publications. Also, using researcher-reported dilemmas (e.g., R1–R3 narratives) presents the risk of subjective bias, as experiences were recalled and selected retrospectively. Innovative as they were, the incorporation of such Eastern philosophical constructs as Karma Yoga and Dharma for empirical validation in research for universality.
